# Dermoscopic features of papillated Bowen’s disease

**DOI:** 10.1016/j.jdcr.2024.06.031

**Published:** 2024-07-08

**Authors:** Jonathan Stevens, Claudia Schroder, Magdalena Delgado

**Affiliations:** a Dermatology Department, Institute of Oncology, Fundación Arturo López Pérez (FALP), Santiago, Chile; b Faculty of Medicine, Department of Dermatology, University of Chile, Santiago, Chile; c Pathology Department, Institute of Oncology, Fundación Arturo López Pérez (FALP), Santiago, Chile

**Keywords:** Bowen's disease, dermoscopy, milky red honeycomb, squamous cell carcinoma, verrucous lesion

## Clinical presentation

**Fig 1:**
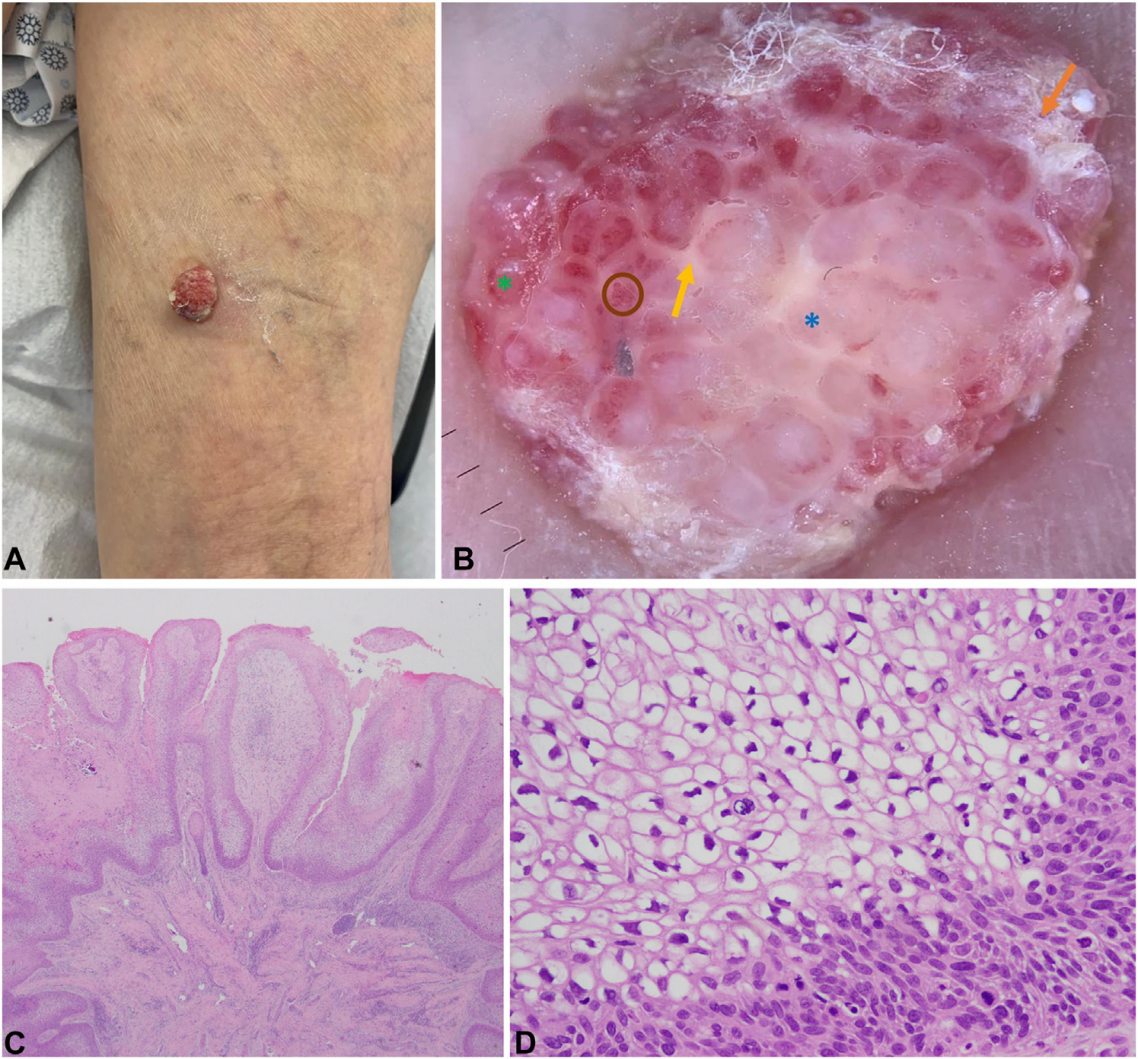
**A,** Macroscopic physical examination: a 10 × 15 mm well-defined symmetrical erythematous tumor in the posterior region of the right thigh. **B,** Dermatoscopic features: *milky red* honeycomb appearance with whitish septa (*yellow arrow*) separating whitish lacunae (*blue asterisk*) in the center and red lacunae on the periphery (*green asterisk*), some with glomerular vessels inside (*brown circle*), with small continuous whitish structureless areas on the periphery (*orange arrow*). **C,** Histopathological examination showing papillomatous exophytic proliferation, consisting of axes of fibroconnective tissue covered by squamous epithelium formed by atypical cells, in a significant proportion with clear cytoplasm (2× magnification). **D,** Atypical squamous intraepithelial proliferation with keratinocytes with clear cytoplasm and well-defined borders, nuclear pleomorphism, and mitosis (40× magnification).

A 90-year-old female with controlled hypertension, presented with a 6-months history of an asymptomatic progressively growing pink lesion in the right thigh. There is no personal or family history of skin cancer. Clinical examination showed a 10 × 15 mm well-defined symmetrical erythematous tumor in the posterior region of the right thigh (Fig 1, *A*).

## Dermatoscopic appearance

Dermoscopy revealed a milky red honeycomb appearance with whitish septa separating whitish lacunae in the center and red lacunae on the periphery, some with glomerular vessels inside, with small continuous whitish structureless areas on the periphery (Fig 1, *B*). Complete surgical removal was performed.

## Histologic diagnosis

Histopathology revealed intraepidermal squamous cell carcinoma, clear cell papillomatous/papillated subtype (Fig 1, *C* and *D*).

**Key message**

Bowen’s disease usually manifests as a slowly enlarging erythematous scaly patch or plaque.^1^ An uncommon variant of Bowen’s disease showing a verrucous appearance with a prominent clear cell change on histopathology was recently reported in 2017.^1^

Given the rarity of this presentation, the differential diagnosis should include pagetoid Bowen's disease, extramammary Paget’s disease, clear cell acanthoma, superficial spreading melanoma, sebaceous carcinoma, and trichilemmal carcinoma.^1^

Detailed descriptions of its dermoscopic features have been quite limited.^2^ There may be some difficulty recognizing the characteristic vascular structures and surface scales of conventional Bowen's disease.^2^ We propose a new dermoscopy feature called “milky red honeycomb”: whitish septa separating whitish lacunae in the center, red lacunae on the periphery with some glomerular vessels inside, and small continuous whitish structureless areas on the periphery.

## Conflicts of interest

None disclosed.
